# New genetic insights into immunotherapy outcomes in gastric cancer via single-cell RNA sequencing and random forest model

**DOI:** 10.1007/s00262-024-03684-8

**Published:** 2024-05-02

**Authors:** Dajun Yu, Jie Yang, BinBin Wang, Zhixiang Li, Kai Wang, Jing Li, Chao Zhu

**Affiliations:** 1 Jinan University, Guangzhou, Guangdong, China; 2 Department of Radiation Oncology, The Second Clinical Medical College (Shenzhen People’s Hospital) of Jinan University, Shenzhen Guangdong, China; 3 Department of Surgical Oncology, The First Affiliated Hospital of Bengbu Medical University, Bengbu, 233000 People’s Republic of China

**Keywords:** Gastric cancer, Immunotherapy, Biomarkers, METTL1, Single-cell RNA sequencing, Random forest model

## Abstract

**Objective:**

The high mortality rate of gastric cancer, traditionally managed through surgery, underscores the urgent need for advanced therapeutic strategies. Despite advancements in treatment modalities, outcomes remain suboptimal, necessitating the identification of novel biomarkers to predict sensitivity to immunotherapy. This study focuses on utilizing single-cell sequencing for gene identification and developing a random forest model to predict immunotherapy sensitivity in gastric cancer patients.

**Methods:**

Differentially expressed genes were identified using single-cell RNA sequencing (scRNA-seq) and gene set enrichment analysis (GESA). A random forest model was constructed based on these genes, and its effectiveness was validated through prognostic analysis. Further, analyses of immune cell infiltration, immune checkpoints, and the random forest model provided deeper insights.

**Results:**

High METTL1 expression was found to correlate with improved survival rates in gastric cancer patients (*P* = 0.042), and the random forest model, based on METTL1 and associated prognostic genes, achieved a significant predictive performance (AUC = 0.863). It showed associations with various immune cell types and negative correlations with CTLA4 and PDCD1 immune checkpoints. Experiments in vitro and in vivo demonstrated that METTL1 enhances gastric cancer cell activity by suppressing T cell proliferation and upregulating CTLA4 and PDCD1.

**Conclusion:**

The random forest model, based on scRNA-seq, shows high predictive value for survival and immunotherapy sensitivity in gastric cancer patients. This study underscores the potential of METTL1 as a biomarker in enhancing the efficacy of gastric cancer immunotherapy.

**Supplementary Information:**

The online version contains supplementary material available at 10.1007/s00262-024-03684-8.

## Introduction

Gastric cancer is universally recognized as a highly fatal malignancy, contributing significantly to global cancer mortality rates [1–3]. Annually, it is responsible for the diagnosis of hundreds of thousands of individuals, frequently at advanced stages of the disease [4, 5]. Despite strides in the early detection and therapeutic approaches for gastric cancer in recent years, substantial obstacles remain [6, 7]. Surgical interventions may offer a measure of success in select instances, yet their effectiveness is limited for patients presenting with advanced or metastatic gastric cancer. Consequently, researchers are dedicated to exploring cutting-edge technologies, novel drug compounds, and advanced genetic and molecular techniques with the potential to revolutionize the way we understand, diagnose, and treat gastric cancer [8].

The advent of immunotherapy marks a pivotal development in the landscape of cancer treatment [9–11], introducing a paradigm that leverages the inherent capacity of a patient's immune system to identify and eradicate cancerous cells. This approach, offering a favorable profile of side effects and the promise of enduring efficacy compared to conventional therapies such as chemotherapy and radiotherapy, represents a significant advancement [12, 13]. However, the variability in immune response and tumor biology across individuals means that immunotherapy does not uniformly benefit all patients with gastric cancer. Thus, there exists a pressing need to devise methods capable of predicting the likelihood of a positive response to immunotherapy among these patients.

Biological markers have been extensively utilized in cancer research to enhance comprehension and forecast the treatment response of patients [14–16]. These markers can provide critical insights into the tumor's biological attributes and the patient's potential reaction to specific treatments. Although several biomarkers have been associated with prognosis and therapeutic response in gastric cancer, the quest for more predictive tools and methodologies continues unabated.

The emergence of single-cell RNA sequencing (scRNA-seq) and random forest modeling as cutting-edge technologies holds the promise of transforming cancer research [17–19]. scRNA-seq offers a granular view of gene expression at the individual cell level, thereby illuminating the heterogeneity present within tumors [20]. Meanwhile, random forest models offer a powerful statistical framework for analyzing vast datasets and producing accurate predictive models [21, 22].

With this context, our study aims to harness the capabilities of scRNA-seq and random forest modeling to unearth novel biomarkers for gastric cancer and to develop a predictive framework capable of forecasting immunotherapeutic responses in gastric cancer patients. We anticipate that such a model will empower clinicians to tailor treatment approaches to individual patient profiles, thereby elevating the likelihood of therapeutic success and enhancing the quality of life for those affected.

## Materials and methods

### Acquisition and preprocessing of scRNA-seq data

ScRNA-seq data were procured, encompassing 42,968 cells derived from 10 samples across 6 patients with gastric cancer diagnoses. This dataset was sourced from the Gene Expression Omnibus (GEO) database (accessible via http://www.ncbi.nlm.nih.gov/geo/) and produced on the Illumina NovaSeq 6000 platform, adhering to a tenfold read depth relative to the genome [23]. For reference, the dataset is cataloged under accession number GSE163558. The R package Seurat, version 4.1.1, was employed for quality assurance, statistical evaluation, and exploratory analysis of the scRNA-seq data.

Quality control measures led to the exclusion of cells not meeting predefined criteria: Cells manifesting fewer than three detected genes, those with under 200 detected genes, and cells exhibiting mitochondrial gene expression below 10% were systematically removed. Subsequently, gene expression data from the filtered cell population were normalized employing a linear regression model. Dimensionality reduction was facilitated through principal component analysis, followed by clustering analysis via the uniform manifold approximation and projection (UMAP) technique, employing the first 15 principal components (PCs). This process set the stage for conducting gene set enrichment analysis (GESA) on differentially expressed genes (DEGs) identified across clusters [24].

### Identification of METTL1-associated diagnostic and prognostic genes

Bulk RNA-seq data for gastric cancer samples (*n* = 433) were retrieved from the GEO database (GSE84437). METTL1-associated genes were pinpointed using the Ingenuity Pathway Analysis (IPA) software, version v01-21-03. Prognostic genes were discerned with the aid of the survival package in R.

### Development of a random forest prognostic model

The prognostic significance of genes with prognosis-related attributes was evaluated by applying the random forest methodology for survival analysis [25]. This machine learning framework is tailored for identifying genes with notable prognostic merit, achieved by constructing many decision trees for classification purposes. These trees were then applied to categorize given input data vectors. The random forest model incorporates bootstrapping and random sample splitting from the original dataset to fabricate each decision tree, setting aside approximately one-third of the samples to form a cross-validation set. The out-of-bag (OOB) samples, serving as a distinct bootstrap testing set, were utilized in the tree construction phase, known as the OOB sample set. Survival analysis employing the random forest approach was instrumental in discerning the prognostic significance of genes, thus facilitating the identification of genes pivotal to the study context. This method acknowledges the influence of any variable on the OOB prediction error.

### Evaluation of predictive accuracy in random forest models

To ascertain whether the risk score could act as an independent prognostic indicator for overall survival (OS) within the context of the GSE84437 study, both univariate and multivariate Cox regression analyses were performed, examining its efficacy singly and in conjunction with other clinical parameters. The “timeROC” package was employed to construct time-dependent receiver operating characteristic (ROC) curves, facilitating the evaluation of the model's predictive performance. The objective behind deploying multi-indicator ROC curves was to determine the specificity associated with the random forest model's forecasts.

### Quantitative examination of tumor microenvironment

Estimation techniques were applied to produce matrices and immune scores to quantify the infiltration of stromal and immune cells within gastric cancer tissues, thus reflecting the tumor's intrinsic condition through expression profiling. Differences in tumor purity, stromal scores, and immune scores between identified high-risk and low-risk cohorts were analyzed using the Wilcoxon rank-sum test. The deconvolution of immune components within tumor infiltrates, leveraging data from the TCGA database, was conducted using the Xibo classification method, yielding significant insights.

### Prediction of immunotherapy response using functional enrichment analysis

In the Tumor Immune Dysfunction and Exclusion (TIDE) project (available at http://tide.dfci.harvard.edu/), a computational model focusing on tumor immune evasion mechanisms is under development, incorporating T cell expression profiles among its variables. The TIDE methodology was utilized to assess the potential efficacy of immune checkpoint blockade therapy in gastric cancer patients.

### Tissue collection

The study enrolled 69 patients who were diagnosed with gastric cancer and were undergoing surgical resection treatment at the First Affiliated Hospital of Bengbu Medical University in 2021. Freshly excised tissues were immediately frozen at − 80 °C for later total RNA extraction. All clinical samples were obtained with informed consent from patients, and collecting and handling samples adhered strictly to ethical guidelines. The study received approval from the Ethics Committee of the First Affiliated Hospital of Bengbu Medical College and was in strict accordance with the *Helsinki Declaration*.

### RNA extraction and qRT-PCR analysis

Total RNA extraction from cultured cell lines was conducted utilizing TRIzol reagent (15596018CN, Thermo Fisher Scientific, Inc, USA), adhering to the guidelines provided by the manufacturer. This process was followed by the conversion of total RNA into complementary DNA (cDNA) via the PrimeScript™ RT reagent Kit with gDNA Eraser (RR047A, BaoRui Medical Biotechnology (Beijing) Co., Ltd, Beijing, China). Quantitative real-time PCR analyses were executed using TB Green® Premix Ex Taq™ (Tli RNaseH Plus) (Cat. No. RR420A, Takara Bio (Beijing) Co. Ltd.) alongside gene-specific primers at a concentration of 0.3 nM, as detailed in Table S1. The quantification of gene expression was relative to the GAPDH gene, serving as an internal control, utilizing the 2^−ΔΔCt^ method for relative quantification [26].

### Cell line culture

The human gastric cancer cell lines employed including AGS (CL-0022; Procell, Wuhan, China), MKN45 (CRL-1739; ATCC, USA), and SNU-216 (CRL-5974; ATCC, USA) were used for this study. Additionally, human gastric mucosal epithelial cells, GES-1, were sourced from BoHui Biotechnology (Guangzhou) Co., Ltd (BH-C051, Guangzhou, China), and mouse gastric cancer cells, MFC (CL-0156; Procell, Wuhan, China), were also included in the study. These cell lines were cultured in Dulbecco's Modified Eagle Medium (11,965,092, Thermo Fisher, USA) supplemented with 100 U/mL penicillin, 100 μg/mL streptomycin (Cat#10,378,016, Gibco, USA), and 10% FBS (10099141C, Gibco, USA). The culture was maintained in a constant temperature incubator at 37 °C with 5% CO_2_ [27].

### Lentivirus transduction

Gene silencing or overexpression in cell lines, alongside the establishment of corresponding control lines, was achieved by applying lentiviral transduction techniques. The sequence for the silent lentivirus is delineated in Table S2. For the generation of the requisite lentivirus, the Phage-puro series plasmid, along with auxiliary plasmids Pspax2 and Pmd2.G, in addition to the pSuper-retro-puro series plasmid and auxiliary plasmids gag/pol and VSVG, was co-transfected into HEK293T cells (CL-0005, Wuhan Puno Sai Life Science Co., Ltd., Hubei, China). This process involved verification, amplification, and purification stages, followed by the production of packaged lentivirus. Plasmid and lentivirus packaging services were furnished by Sangon Biotech, Shanghai, China.

Lentiviral infection was initiated by seeding 1 × 10^5^ AGS or MCF cells into each well of a 6-well plate. Upon achieving 60–70% confluency, the cell medium was enriched with a designated quantity of packaged lentivirus (MOI = 10, effective titer approximating 1 × 106 TU/mL) and 5 μg/mL polybrene (TR-1003, Merck, USA) to facilitate transfection. Four hours post-transfection, the medium containing polybrene was diluted by adding an equivalent volume of medium. The medium was refreshed 24 h following transfection. Selection of resistant cells using 1 μg/mL puromycin (A1113803, Thermo Fisher, USA) commenced 48 h post-transfection, culminating in the establishment of stable AGS or MCF cell lines [28].

### CCK-8 assay

Cell viability was quantified in cells at the logarithmic growth phase, seeded at a density of 1 × 10^4^ cells per well in a 96-well plate, and incubated overnight. The evaluation employed the CCK-8 assay kit (E606335, Sangon Biotech, China) as follows: CCK-8 reagent (10 μL) was introduced to each well at intervals of 0, 24, 48, and 72 h during cell culture. Following a 1-h incubation in a humidified incubator at 37 °C, absorbance at 450 nm was measured with an Epoch Microplate Spectrophotometer (Bio-Tek, USA) [29].

### Apoptosis detection via flow cytometry

The Annexin V-FITC/PI double staining method was employed for apoptosis detection. T98G or LN-229 glioblastoma cells were collected, centrifuged at 800g, and discarded supernatant. After two PBS washes, cells were resuspended in 500 μL of binding buffer as per the cell apoptosis detection kit's protocol (556,547, BD Bioscience, USA). To each sample, 5 μL of FITC and 5 μL of PI were added and mixed thoroughly. Post a 15-min incubation, apoptosis was detected using a BD FACSCalibur flow cytometer (BD FACSVerse, USA) [30], with Annexin V-FITC indicating positively stained apoptotic cells.

### Isolation and enrichment of CD4^+^ T cells

Peripheral blood mononuclear cells (PBMCs) were initially segregated from the blood of volunteer donors. These cells underwent enrichment for CD4^+^ T cells utilizing the Human CD4^+^ T Cell Enrichment Kit (8802-6831-74, Thermo Fisher, USA). Post-enrichment, the cells were processed through centrifugation over a non-continuous Percoll gradient, specifically layering a 40% Percoll solution atop an 80% solution and centrifuging at 320 g for 25 min. The purified cells were then propagated in RPMI 1640 medium, enriched with 10% FBS, 1% antibiotics, 10 mM HEPES, 2 mM L-glutamine, 1 mM sodium pyruvate, MEM non-essential amino acids, and 55 μM β-mercaptoethanol [31, 32].

### Co-culture of immune cells and cancer cells

CD4^+^ T cells derived from healthy volunteers were co-cultivated with gastric cancer cells. The T cells were initially labeled with CFSE (C34554, Thermo Fisher, USA) diluted at a 1:1000 ratio and then mixed gently. After a 24-h co-cultivation period with gastric cancer cells, the T cells were collected, and the CFSE intensity was quantified via flow cytometry. The T cells were then fixed, permeabilized, and stained with an anti-Ki-67 antibody (ab279653, Abcam, UK) for proliferation assessment through flow cytometric analysis using a BD FACS Canto II system [33, 34].

### Immunofluorescence staining

Cells were affixed onto glass slides with 4% formaldehyde for 10 min and permeabilized using 0.1% Triton X-100 (93,443, Sigma-Aldrich, USA). They were incubated overnight at 4 °C with a METTL1 antibody (ab271063, Abcam, USA) at a 1:100 dilution. Following this, cells were incubated with a FITC-labeled secondary antibody (Alexa Fluor 488) (ab150077, Abcam, UK) diluted 1:1000 for 1 h. Nuclei staining was performed using DAPI. Fluorescence microscopy (Olympus, Japan) was employed to observe and capture the fluorescence intensity across coverslips, with quantitative analysis conducted using ImageJ software (National Institutes of Health) [35].

### Western blot

Total protein from cells was extracted employing RIPA lysis buffer (P0013C, BiYunTian, Shanghai, China) supplemented with PMSF. The mixture was incubated on ice at 4 °C for 30 min and subsequently centrifuged at 8000g for 10 min to separate the supernatant. Protein concentration within the supernatant was ascertained using the BCA assay kit (Catalog number 23227, Thermo Fisher, USA). A quantity of 50 μg of protein was mixed with 2 × SDS loading buffer and heated at 100 °C for 5 min. The prepared samples were then subjected to SDS-PAGE gel electrophoresis, followed by protein transfer onto a PVDF membrane. The membrane was blocked using 5% skim milk at ambient temperature for 1 h before overnight incubation at 4 °C with primary antibodies diluted in the buffer: CTLA4 (ab237712, 1:2500, Abcam, UK), PDCD1 (ab309363, 1:2500, Abcam, UK), and GAPDH (ab9485, 1:2500, Abcam, UK) serving as the loading control. The membrane was washed thrice with TBST, each wash lasting 10 min, and then incubated with HRP-conjugated secondary antibody, goat anti-rabbit IgG H&L (HRP) (ab97051, 1:2000, Abcam, UK) for 1 h. After additional washes with TBST, the membrane was positioned on a clean glass plate for detection. Components A and B from the ECL fluorescence detection kit (catalog number abs920, Shanghai ABclonal Technology Co., Ltd., Shanghai, China) were mixed in equal volumes under dim light and applied to the membrane. Imaging was carried out using the Bio-Rad imaging system (Bio-Rad, USA), and the images obtained were analyzed with Quantity One v4.6.2 software. The relative abundance of proteins was expressed through the ratio of the grayscale intensity of the target protein bands to that of the GAPDH band [36].

### Animal experiments

Male BALB/c mice, aged 4–6 weeks, were acquired from Beijing Vital River Laboratory Animal Technology Co., Ltd. (Beijing, China). Seventy-two mice were housed in standard cages under sterile conditions and maintained at a constant room temperature of 23 ± 1 °C. The mice were exposed to a 12-h light–dark cycle with controlled temperature and humidity. They were provided ad libitum access to food and water and were acclimatized for one week before the experiment. The experimental procedures and animal use protocols in this study were conducted following international ethical guidelines for animal experimentation and received approval from the Animal Ethics Committee of the First Affiliated Hospital of Bengbu Medical College.

We utilized the MFC cell line derived from mouse gastric cancer to establish a mouse gastric cancer model. Initially, we assessed the effectiveness of lentiviral infection. The expression of METTL1 in MFC cells was detected using the RT-qPCR method. The findings demonstrated a reduction in METTL1 expression in the sh-METTL1 group compared to the sh-NC group. Notably, the silencing efficiency of sh-METTL1-1 was higher than that of sh-METTL1-2. Consequently, sh-METTL1-1 was selected for silencing MFC cells in subsequent experiments. Conversely, the overexpression of METTL1 led to a substantial increase in METTL1 expression in MFC cells (Figure S1).

MFC cells, numbering 1 × 10^6^, were suspended in 100 μl of serum-free culture medium and subsequently implanted subcutaneously into mice to facilitate tumor development. The growth and localization of the tumors were monitored by measuring subcutaneous nodules with a caliper at 7-day intervals. Both the longitudinal and transverse diameters of each tumor were measured, with the volume being calculated using the formula V = π/6 × L (long diameter) × W2 (short diameter). Following a 28-day period, the mice were euthanized, and the tumors were excised, photographed, and weighed utilizing an electronic scale. Documentation of experimental data was meticulously conducted. For subsequent immunohistochemical analysis to confirm METTL1 expression within the tumor tissues, the tumors were preserved in cryovials and stored at − 80 °C.

In vivo fluorescence imaging was performed to examine the impact of METTL1 on tumor growth using the In-Vivo Imaging System (Caliper Lifesciences, USA). The study encompassed four groups of mice, each with distinct cellular transfections: sh-NC (MFC cells transfected with sh-NC and injected subcutaneously), sh-METTL1 (MFC cells transfected with sh-METTL1 and injected subcutaneously), oe-NC (MFC cells transfected with oe-NC and injected subcutaneously), and oe-METTL1 (MFC cells transfected with oe-METTL1 and injected subcutaneously). Each specified group consisted of six mice.

After subcutaneously injecting cancer cells using the method described above, we administer CTLA4 or PD1 inhibitors for immunotherapy once the tumor volume reaches 100 mm3 [37, 38]. A volume of 100 μl of serum-free medium was used to add 200 μg of anti-CTLA4 (ab237712, Abcam, Cambridge, UK) and anti-PD-1 (ab214421, Abcam, Cambridge, UK) per mouse to achieve the blockade of CTLA4 and PD-1 for immunotherapy. Anti-IgG (ab172730, Abcam, Cambridge, UK) was used as a control [38]. Survival rate analysis was conducted on mice. Animal grouping: sh-NC + anti-IgG (subcutaneous injection of sh-NC transfected MFC cells + intraperitoneal injection of anti-IgG), sh-METTL1 + anti-IgG (subcutaneous injection of sh-METTL1 transfected MFC cells + intraperitoneal injection of anti-IgG), sh-METTL1 + anti-CTL4 (subcutaneous injection of sh-METTL1 transfected MFC cells + intraperitoneal injection of anti-CTL4), sh-METTL1 + anti-PD1 (subcutaneous injection of sh-METTL1 transfected MFC cells + intraperitoneal injection of anti-PD1), oe-NC + anti-IgG (subcutaneous injection of oe-NC transfected MFC cells + intraperitoneal injection of anti-IgG), oe-METTL1 + anti-IgG (subcutaneous injection of oe-METTL1 transfected MFC cells + intraperitoneal injection of anti-IgG), oe-METTL1 + anti-CTL4 (subcutaneous injection of oe-METTL1 transfected MFC cells + intraperitoneal injection of anti-CTL4), and oe-METTL1 + anti-PD1 (subcutaneous injection of oe-METTL1 transfected MFC cells + intraperitoneal injection of anti-PD1). Each group comprised six mice.

### In vivo fluorescence imaging

Prior to euthanasia, mice were subjected to in vivo fluorescence imaging through the intraperitoneal administration of 2 mL of fluorescein (150 mg/mL) provided by Caliper Lifesciences, USA. The imaging process utilized an in vivo imaging system from the same manufacturer, with live image software facilitating the analysis. During imaging, the mice were anesthetized using 2% isoflurane and captured by a cooled CCD camera. A quantitative assessment of the bioluminescent signals emitted by the xenograft-bearing mice was performed using Vivo Image 3.0 software [39, 40].

### Immunohistochemistry staining

Transplanted tissues were fixed in 10% formalin solution and then dewaxed with xylene in two 10-min sessions. Hydration was achieved through sequential ethanol–water gradients of 100%, 95%, 75%, and 50%, each stage lasting 5 min. Afterward, samples were incubated with H2O2 for 10 min at room temperature. They were then treated with 0.01mol/L citrate buffer, followed by microwave-assisted antigen retrieval for 20 min. Normal goat serum was applied for 5 min at room temperature before the addition of primary antibodies against METTL1 (ab271063, Abcam, USA) and PD-L1 (ab205921, Abcam, USA) overnight at 4 °C. After incubation at 37 °C for 1 h with biotinylated goat anti-rabbit secondary antibody (ab150077, Abcam, USA) for 30 min, DAB chromogenic solution was applied for 1–2 min, followed by hematoxylin counterstaining. The samples were dehydrated and mounted. Optical microscopy was used to observe and capture images from five randomly selected high-magnification fields. Positive staining was determined by the presence of brown or yellow cytoplasm, and the proportion of positively stained cells was quantified [41].

### Statistical analysis

The data utilized in this study have undergone meticulous quality control and preprocessing. The scRNA-seq data were standardized and dimensionally reduced using PCA and UMAP. The Wilcoxon rank-sum test assessed differences among DEGs. IPA software was used to identify METTL1-associated genes from RNA-seq data retrieved from the GEO database. Survival analysis was conducted using the survival package, and random forest models were developed using machine learning to construct and validate decision trees, including OOB sample evaluation. The Cox regression model was employed for survival analysis in both univariate and multivariate formats. The model's efficacy was gauged through time-dependent ROC curves. The TIDE method assessed immunotherapy responses, with significance set at a *p*-value of less than 0.05. Statistical analyses were performed in R version 4.1.1, employing GraphPad Prism 9 for data visualization. Measurement data were presented as mean ± SD, with the unpaired Student's t test for two-group comparisons and one-way ANOVA with a post hoc Tukey test for multiple-group comparisons. A *p*-value of < 0.05 was considered indicative of statistical significance.

## Results

### scRNA-seq of gastric cancer reveals distinct subpopulations of cells specific to the tumor

**Fig. 1:**
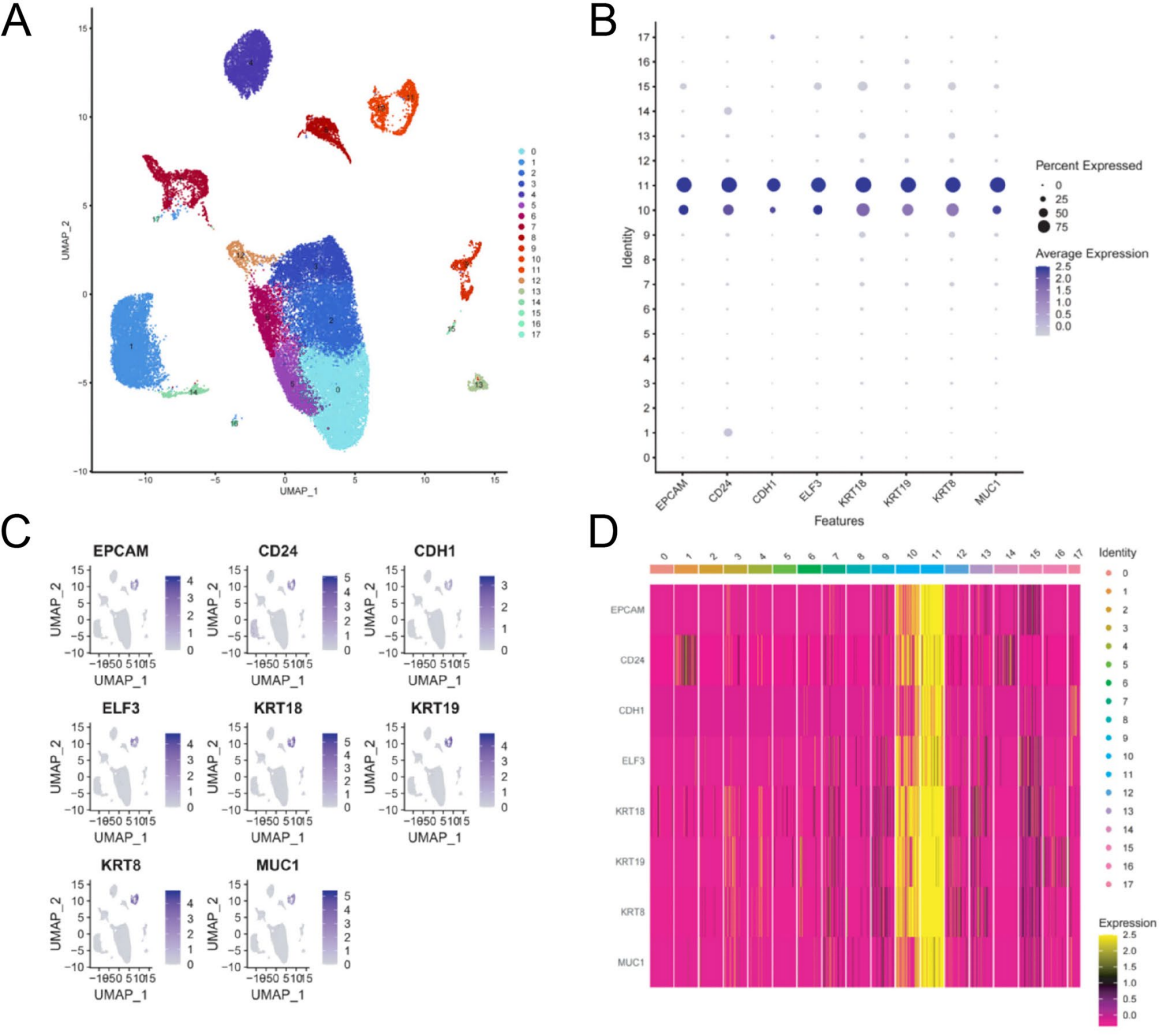
Cell subtypes and expression distribution of key tumor-related genes revealed by scRNA-seq of gastric cancer. **A** Single-cell sequencing divided gastric cancer tissue into 18 subgroups. **B** Differential expression of different genes in 10/11 subgroups. **C** Location of these DEGs. **D** Heatmap of gene expression differences

To ensure the reliability of the data sources, we initially analyzed the results of gastric cancer-related scRNA-seq data obtained from the GEO database (https://www.ncbi.nlm.nih.gov/geo/). Our analysis results indicate that the scRNA-seq data can be categorized into 18 groups (Fig. 1A). The high expression of tumor-related genes (EPCAM, CD24, CDH1, ELF3, KRT18, KRT19, KRT8, and MUC1) in groups 10 and 11 suggests that cells in these groups are tumor cells (Fig. 1B). Figure 1C-D displays the scatter plot and heat map.

### The disparities in gene expression between tumors and normal tissues elucidate the involvement of the tRNA signaling pathway and underscore the significance of METTL1

**Fig. 2:**
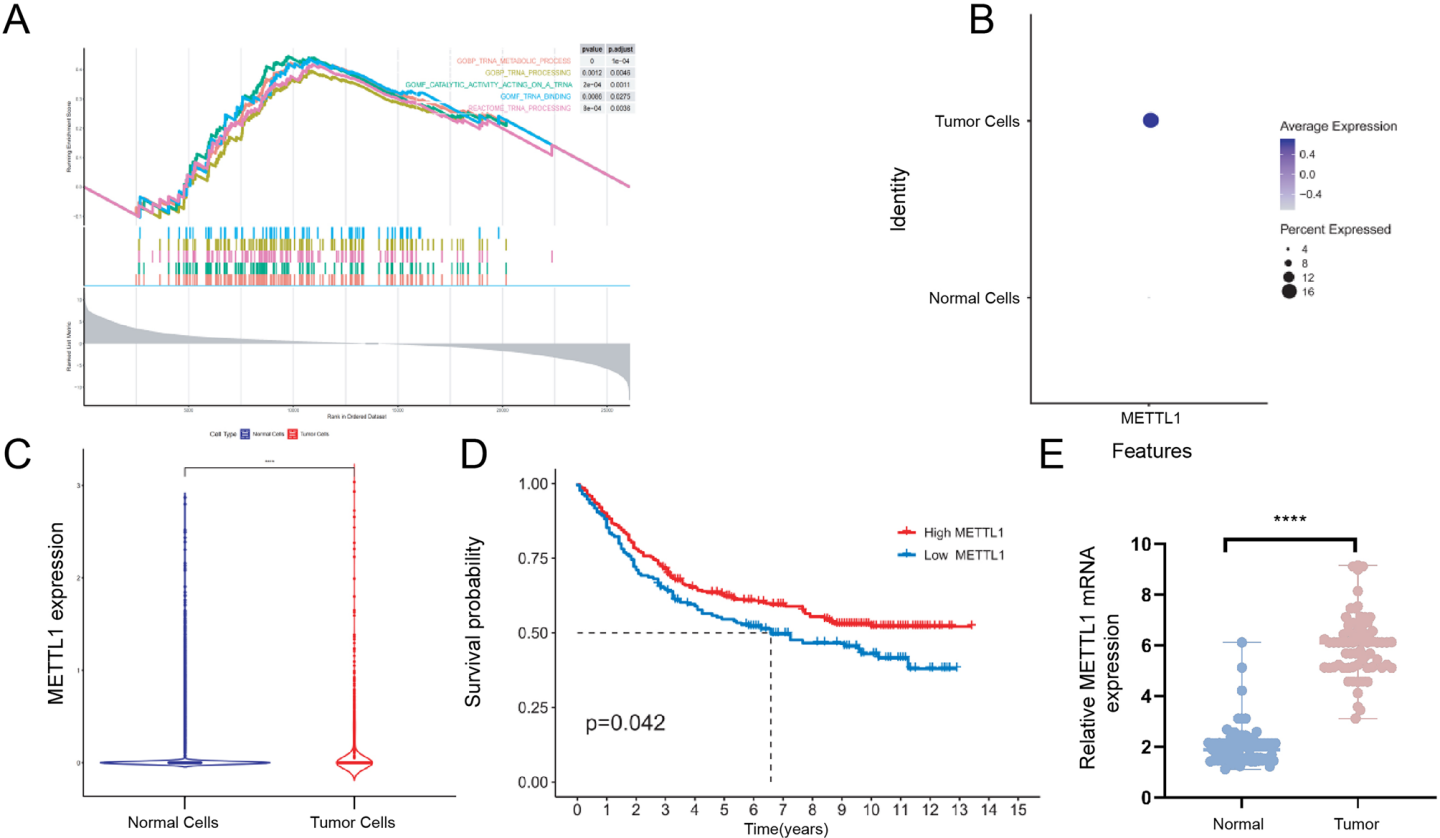
Differential activity of tRNA signaling pathway and association of METTL1 expression with survival in tumor and normal tissues. **A** Gene set enrichment analysis shows differential expression in tRNA-related pathways. **B** Higher expression of METTL1 in gastric cancer cells. **C** Differential expression of METTL1 between tumor and normal cells. **D** Survival curves for METTL1 at different expression levels. **E** qRT-PCR detection of METTL1 expression in tumor and adjacent samples from 69 gastric cancer patients; **p < 0.01; ****p < 0.0001

To further identify genes exhibiting abnormal expression in tumors, we employed GESA to compare the differences between tumor and normal tissues. Our findings revealed that the tRNA-related signaling pathway displays increased activity in tumor tissues compared to normal tissues (Fig. 2A). Additionally, we found that the METTL1 gene was overexpressed in tumor tissues of GSE84437 (Fig. 2B-C). Survival analysis revealed that higher expression of METTL1 was associated with improved patient survival compared to lower expression (Fig. 2D). Furthermore, we observed an overexpression of METTL1 in gastric cancer tissue samples obtained from 69 patients with gastric cancer (Fig. 2E).

### Random forest model based on genes related to METTL1 demonstrate potential for prognostic analysis

**Fig. 3:**
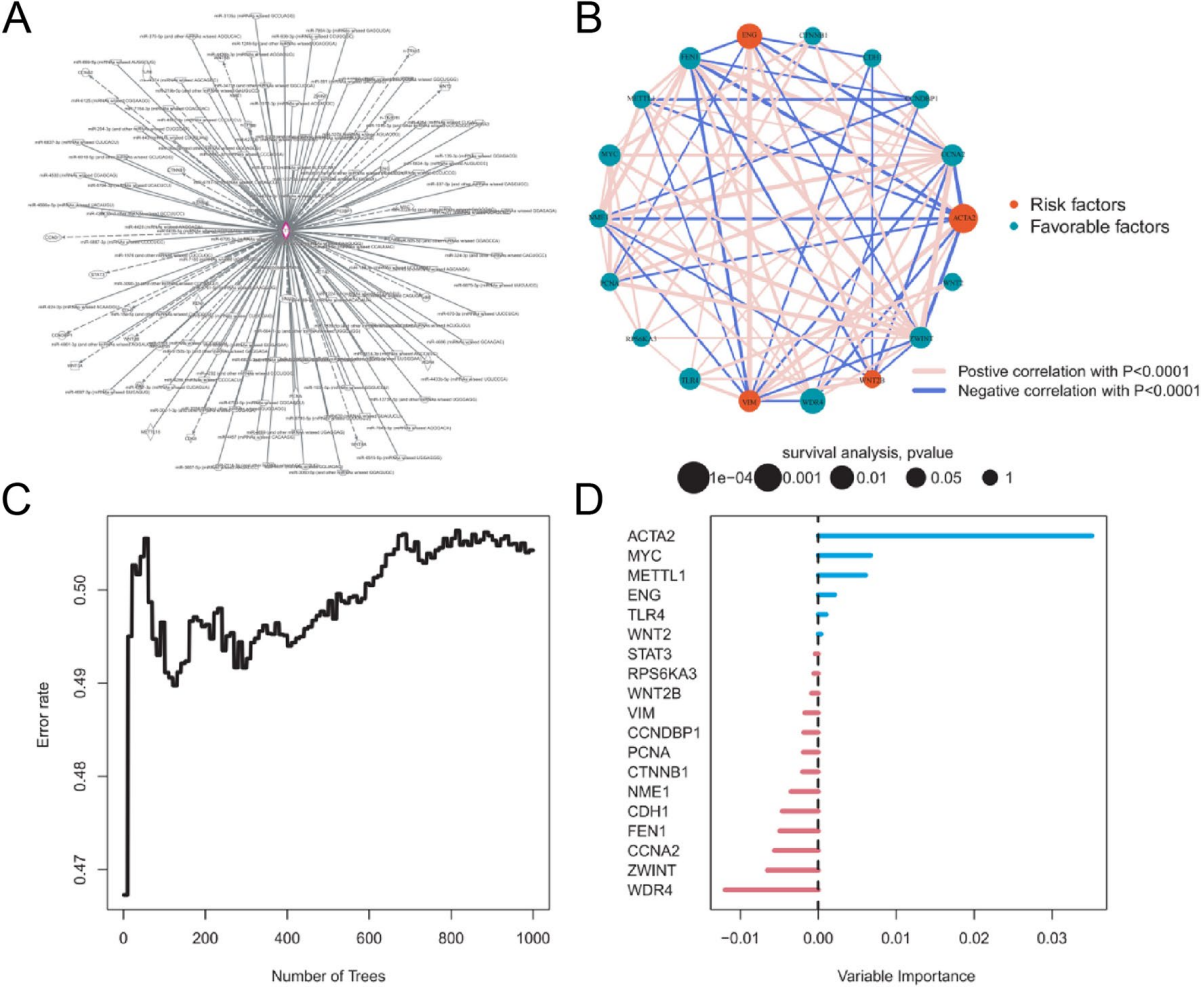
Network analysis of METTL1 and related prognostic genes, and predictive evaluation of random forest model. **A** Network of METTL1 and its associated genes. **B** Network of related prognostic genes. **C** OOB prediction error rate for each tree generated in the random forest model. **D** Feature prognostic gene characteristics: ranking of prognostic genes based on their value. Genes highlighted in red indicate their ability to improve prediction accuracy, while those in blue indicate the opposite

To further elucidate the potential regulatory mechanisms, we identified genes associated with mettl1 using a novel technique called Intuitive Pathway Analysis (IPA) (Fig. 3A). We selected prognostic genes and visualized them in a network diagram (Fig. 3B). Subsequently, we included the pertinent prognostic genes in a random forest survival model (Fig. 3C). Furthermore, we identified ACTA2, MYC, METTL1, ENG, TLR4, and WNT2 as factors that enhance the model's accuracy. Conversely, STATA3, RPS6KA3, WNT2B, VIM, CCNDBP1, PCNA, CTNNB1, NME1, CDH1, FEN1, CCNA2, ZWINT, and WDR4 were shown to exhibit an inverse relationship (Fig. 3D).

### The risk scoring system demonstrates the disparity in survival rates between high-risk and low-risk patients with gastric cancer

**Fig. 4:**
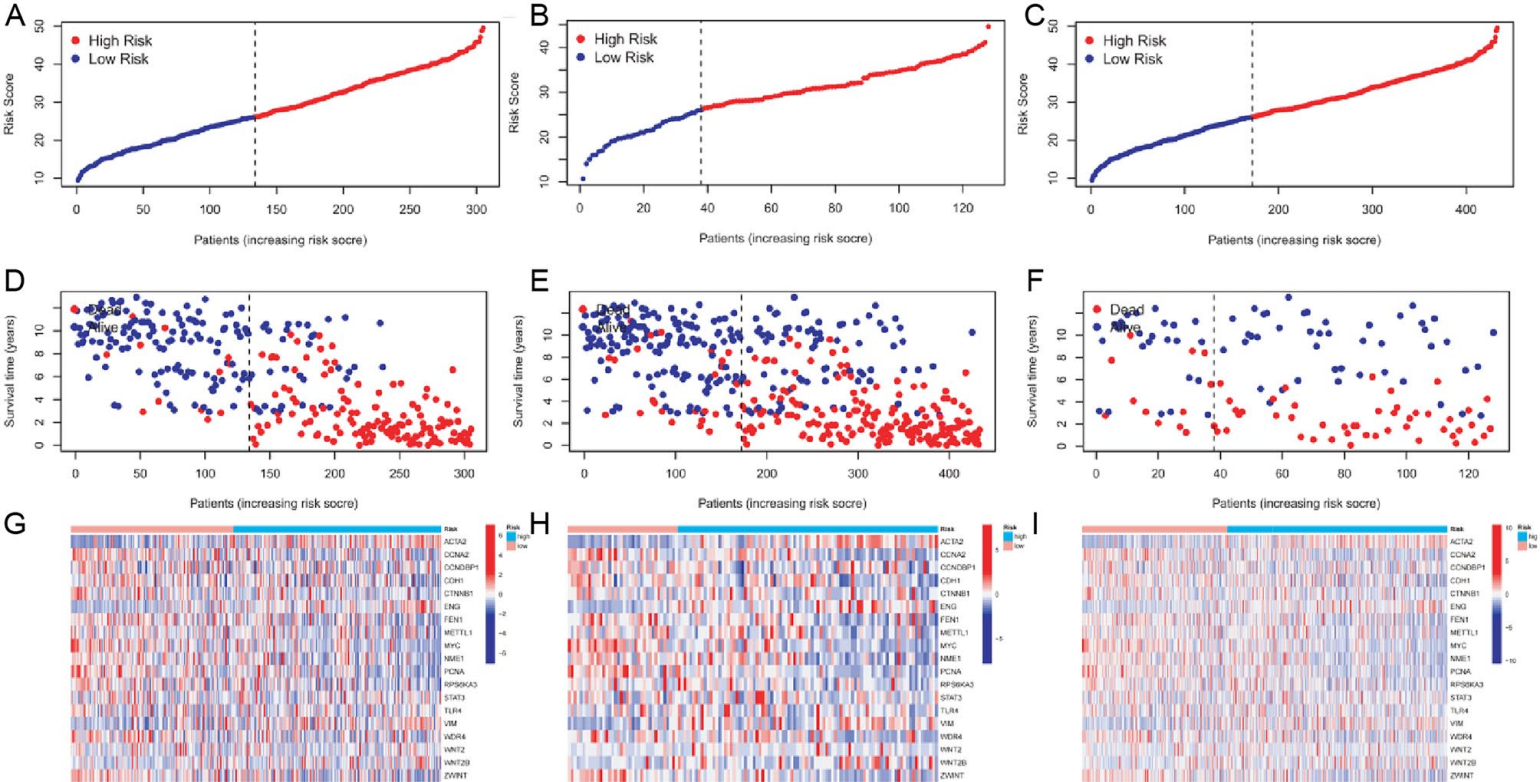
Score distribution, survival status, and heatmap of prognostic gene expression in subgroups of gastric cancer patients under the risk scoring system. **A** Distribution and median of risk scores for the training group, **B** testing group, and **C** entire group of patients. **D** OS status, OS, and risk score distribution for the training group, **E** testing group, and **F** all patients. Heatmap of prognostic genes for the training group (**G**), testing group (**H**), and high-risk and low-risk patients (**I**)

**Fig. 5:**
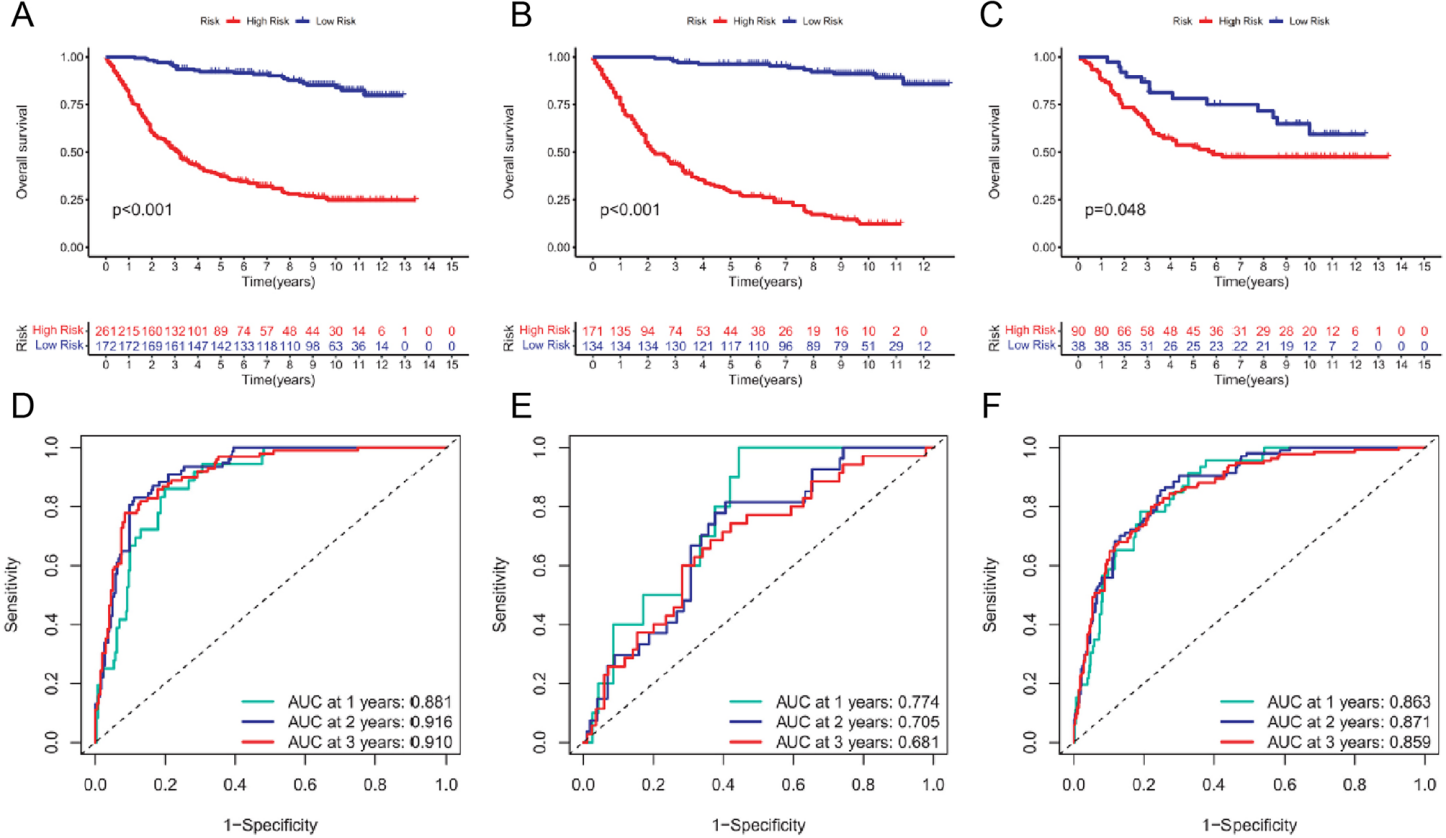
Survival analysis and ROC validation of high-risk and low-risk subgroups of gastric cancer patients. **A** Survival analysis for the training group, **B** testing group, and **C** entire group of patients. **D** AUC values for the training group (1-, 2-, 3-AUC = 0.881, 0.916, 0.910), **E** testing group (1-, 2-, 3-AUC = 0.774, 0.705, 0.681), and **F** all patients (1-, 2-, 3-AUC = 0.863, 0.871, 0.859)

To facilitate the study of the model, the patients were divided into high-risk and low-risk groups using the optimal threshold obtained from the training set (Fig. 4A). The identical method was also utilized in the testing group (Fig. 4B) and the complete patient population (Fig. 4C). In comparison to patients with a lower mortality risk in all categories, individuals with a greater risk of death exhibit elevated mortality rates (Fig. 4D-F). A heatmap illustrating gene expression changes associated with prognosis in high-risk and low-risk patients was generated and presented (Fig. 4G-I). Furthermore, the survival analysis demonstrated that high-risk patients exhibit inferior overall survival compared to low-risk patients, irrespective of their assigned group (Fig. 5A-C). The ROC curves for each group were analyzed. For the training group, the AUC values were as follows: 1-AUC = 0.881, 2-AUC = 0.916, 3-AUC = 0.910. Similarly, for the testing group, the AUC values were 1-AUC = 0.774, 2-AUC = 0.705, and 3-AUC = 0.681. Lastly, for the total patient group, the AUC values were: 1-AUC = 0.863, 2-AUC = 0.871, and 3-AUC = 0.859. These results are presented in Fig. 5D-F.

### Validation of the risk score as an independent prognostic factor for gastric cancer

**Fig. 6:**
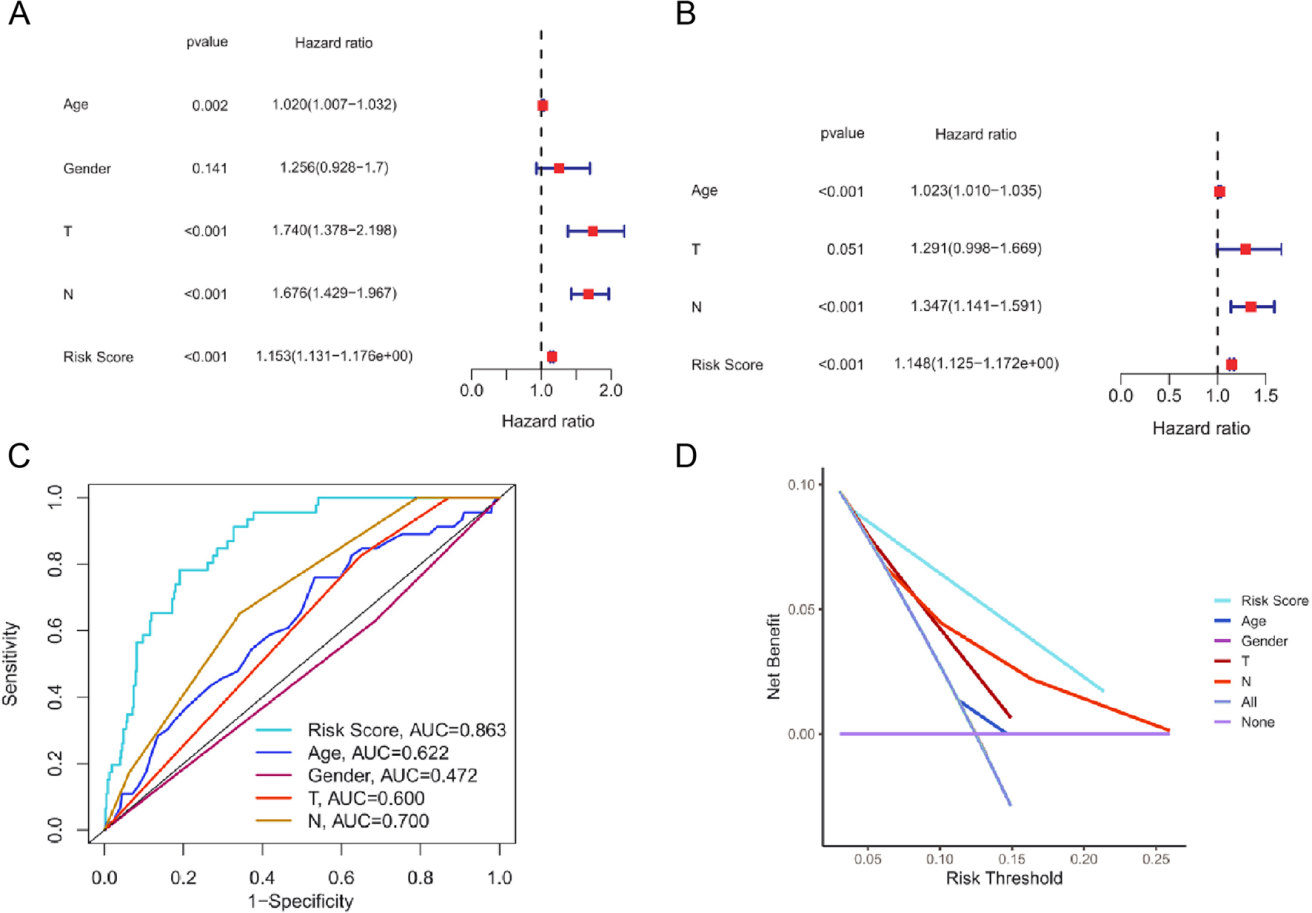
Statistical validation of risk scoring as an independent prognostic factor for gastric cancer and comparison of model prediction performance. **A** Univariate analysis of risk scoring. **B** Multivariate Cox analysis of risk scoring. **C** Multi-index ROC curve. **D** Decision curve analysis of clinical features and risk model

We performed Cox regression analysis to determine if the predictive accuracy of our model for patient prognosis is affected by other variables that may influence clinical outcomes. In this analysis, the model was tested in conjunction with these additional parameters. The results suggested that the risk score was considered an independent prognostic factor, regardless of the impact of other clinical variables (Fig. 6A-B). The results of the multivariate AUC analysis demonstrated that the random forest column line chart analysis (AUC = 0.863) had a superior prognostic value compared to the AUC of other factors (Fig. 6C). Furthermore, decision curve analysis demonstrated that random forest analysis provides more accurate predictions for gastric cancer patients than line graphs (Fig. 6D).

### A comprehensive analysis of the immune microenvironment: investigating the relationship between risk score and infiltration of immune cells

To comprehend the correlation between immune function and risk scoring, we initially compared the outcomes of various groups (Figure S2A). Subsequently, we utilized estimation software along with estimation algorithms to evaluate the immunological scores, stromal scores, and estimation scores of each patient, which indicate the immune microenvironment of gastric cancer. The results indicated that the interstitial score, immune score, and estimated score were comparatively high in high-risk patients (Figure S2B). Moreover, we employed the Sibar classification method to quantitatively assess the extent of immune cell infiltration in gastric cancer samples. This result enabled us to calculate the relative abundance of immune cells in the tumor microenvironment. The infiltration of immune cells varied significantly, including B memory cells, plasma cells, CD4 memory-activated T regulatory cells (Treg), resting macrophages, and resting dendritic cells (Figure S2C).

### Analysis of the association between risk scoring and immunotherapy response sensitivity in gastric cancer

We are interested in investigating the relationship between risk score and immunotherapy response because of the correlation between risk score and immune cell infiltration. The research showed that the high-risk group exhibited higher TIDE values (Figure S3A). Furthermore, patients in the high-risk group demonstrated decreased sensitivity to immunotherapy compared to those in the low-risk group (Figure S3B). Moreover, the risk score negatively correlated with the expression levels of immune checkpoint genes, namely CTLA4, CD274, PDCD1, CD80, LGALS9, and LAG3, as depicted in Figure S3C.

### Regulatory function of METTL1 in gastric cancer cells and its influence on the immune response

To further validate the findings of the bioinformatics analysis regarding the regulation of gastric cancer immunotherapy by the METTL1 gene, we performed supplementary in vitro cell experiments for verification.

**Fig. 7:**
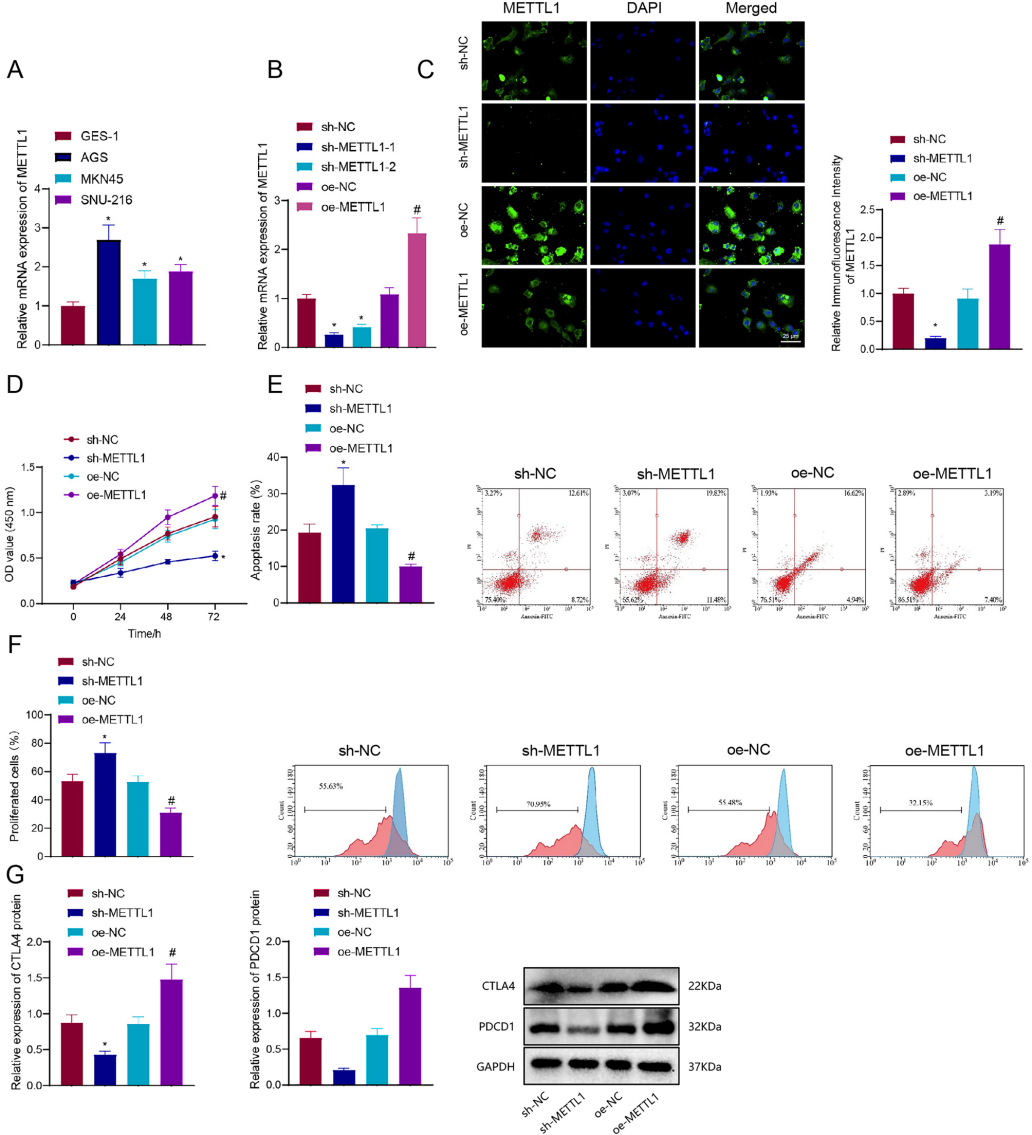
In vitro cell experiments to verify the regulatory role of METTL1 in gastric cancer cells and its impact on immune response. **A** RT-qPCR detection of METTL1 expression levels in human gastric epithelial cells GES-1 and gastric cancer cell lines AGS, MKN45, and SNU-216. **B** RT-qPCR detection of METTL1 expression and silencing efficiency in AGS cell lines. **C** Immunofluorescence detection of METTL1 protein expression in AGS cells (scale bar: 25 μm; DAPI: Blue; METTL1: Green, nucleus). **D** CCK-8 assay to measure cell proliferation in AGS cell lines. **E** Flow cytometry to assess apoptosis in AGS cell lines. **F** CFSE proliferation assay to evaluate the proliferation capacity of T cells co-cultured with AGS cell lines. **G** Western blot to detect the protein expression levels of CTLA4 and PDCD1 in AGS cells. *P < 0.05 compared with the sh-NC group; # *P* < 0.05 compared with the oe-NC group; Cell experiments were repeated three times

We initially detected the expression levels of METTL1 in human gastric mucosal epithelial cells (GES-1) and gastric cancer cell lines (AGS, MKN45, and SNU-216) using RT-qPCR. The results displayed in Fig. 7A revealed an increase in METTL1 expression in AGS, MKN45, and SNU-216 cells when compared to GES-1 cells. Notably, the observed change in AGS cells was more prominent than in MKN45 and SNU-216 cells. Consequently, AGS cells were selected for further functional studies.

Subsequently, METTL1-specific shRNA was utilized to silence the gene through lentivirus infection. To confirm the efficiency of the lentivirus infection, RT-qPCR was employed to assess the expression of METTL1 in AGS cells. The results demonstrated a reduction in the expression of METTL1 in the sh-METTL1 group compared to the sh-NC group in AGS cells. Moreover, the silencing efficiency of sh-METTL1-1 was notably higher than that of sh-METTL1-2. Thus, sh-METTL1-1 was chosen for subsequent gene silencing experiments.

On the contrary, the overexpression of METTL1 led to an increase in METTL1 expression in these cells (Fig. 7B). Immunofluorescence detection was employed to examine the expression of the METTL1 protein in different groups of AGS cells. The results revealed that cells in the oe-METTL1 group exhibited a higher signal intensity of the METTL1 protein in immunofluorescence staining, indicating an upregulation of METTL1 expression (Fig. 7C). Hence, the plasmids acquired via lentiviral infection can be utilized for subsequent experiments.

Next, the CCK-8 assay investigated the correlation between METTL1 expression and cell proliferation. The results demonstrated that the sh-METTL1 group exhibited reduced cell proliferation capacity in AGS cells compared to the sh-NC group. Conversely, the overexpression of METTL1 enhanced the proliferation ability of these cells (Fig. 7D). Moreover, we conducted flow cytometry experiments to investigate the apoptosis rate changes in AGS cells under the influence of METTL1. The results indicated that the sh-METTL1 group exhibited an elevated apoptosis rate compared to the sh-NC group. Conversely, the overexpression of METTL1 decreased the apoptosis rate of gastric cancer cells (Fig. 7E).

We conducted a co-culture experiment of AGS cells with T cells to investigate and confirm the regulatory mechanism of METTL1 on gastric cancer cells. The proliferative capacity of T cells co-cultured with AGS cells was assessed using a CFSE proliferation assay. The findings revealed that when METTL1 expression was suppressed, T cells exhibited enhanced proliferation when co-cultured with AGS cells. Conversely, the opposite effect was observed upon overexpression of METTL1 (Fig. 7F).

Previous research has demonstrated that CTLA4 and PDCD1 are biomarkers with high expression levels in gastric cancer tissues [42]. The low expression level of it can serve as a molecular-level detection standard for successful immunotherapy in patients [43]. Therefore, we conducted a Western blot to analyze the protein expression levels of immune checkpoint proteins CTLA4 and PDCD1. This investigation aimed to determine the impact of METTL1 expression on immunotherapy in clinical settings. The results indicated that silencing METTL1 decreased the protein expression levels of CTLA4 and PDCD1, whereas overexpressing METTL1 increased the levels of both proteins (Fig. 7G).

In conclusion, the overexpression of METTL1 in AGS cells leads to a decrease in T cell activity when co-cultured with AGS cells. This overexpression also enhances the expression of immune checkpoint proteins, CTLA4 and PDCD1, ultimately inhibiting immunotherapy targeting gastric cancer cells.

### Effects of METTL1 in a mouse model and its impact on in vivo immunotherapy response.

To validate the regulatory role of METTL1 in gastric cancer cells and its impact on the immune response, a mouse gastric cancer model was successfully developed using MFC cells that were either overexpressing or silenced for METTL1. Furthermore, we apply the model to treating immune checkpoint inhibitors such as CTLA4 or PD1.

**Fig. 8:**
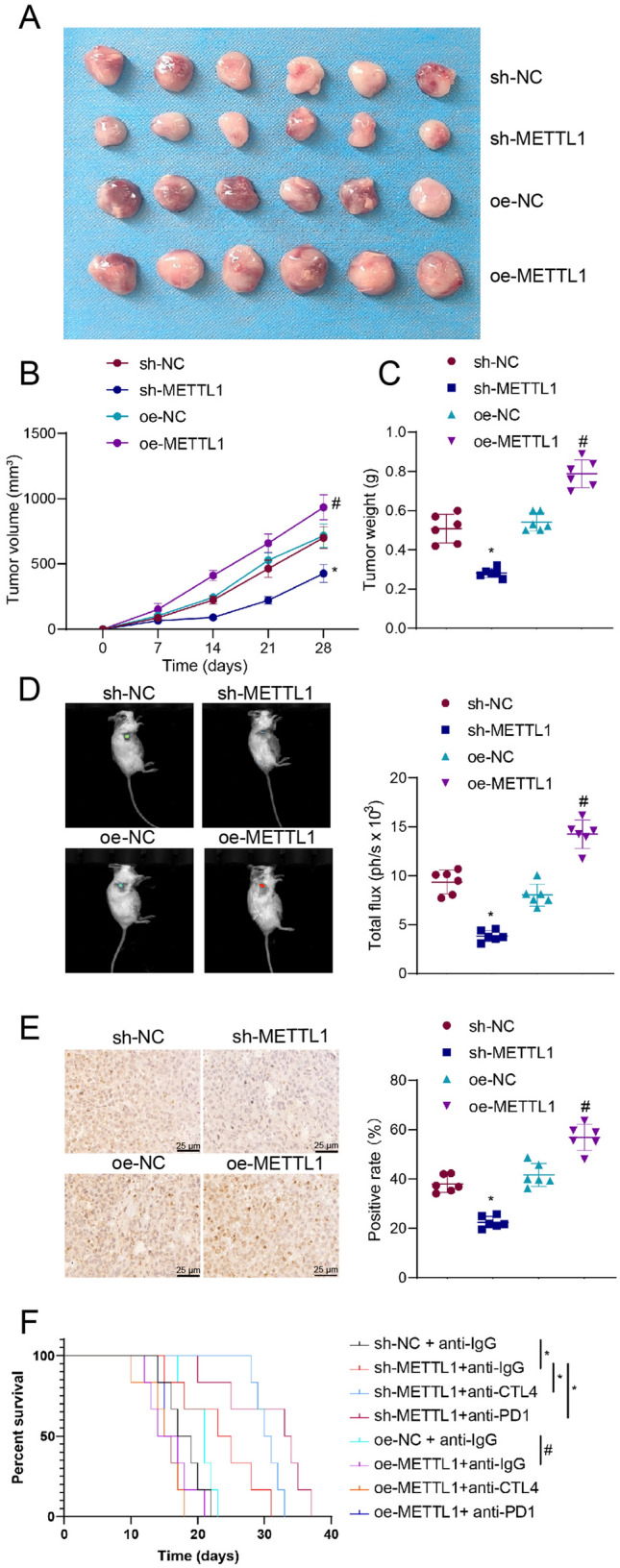
In vivo animal experiments to validate the in vivo effects of METTL1 in a mouse model and its impact on immunotherapy response. **A** Tumor images of mice in each group. **B** Tumor volume changes in each group of mice. **C** Tumor mass in each group. **D** IVIS fluorescence imaging to detect tumor tissue in mice. **E** Immunohistochemistry to detect positive expression of METTL1 in tumor tissue (Scale bar = 25 μm). **F** Kaplan–Meier curve to measure survival period in mice. *P < 0.05 compared with the sh-NC group or sh-NC + anti-IgG group; **P < 0.01 compared with the sh-NC + anti-IgG group; # *P* < 0.05 compared with the oe-NC group or oe-NC + anti-IgG group; 6 mice per group

Firstly, the changes in tumor volume and mass in the different groups of mice show that the sh-METTL1 group had smaller tumor volume and mass compared to the sh-NC group, while the oe-METTL1 group had larger tumor volume and mass compared to the oe-NC group mice (Fig. 8A-C). Subsequently, we used the In-Vivo Imaging System to conduct in vivo fluorescence imaging for detecting tumor formation in mice. The results revealed that the sh-METTL1 mice group displayed lower signal intensity on the fluorescence images, indicating a decrease in tumor growth activity compared to the sh-NC group. Conversely, the oe-METTL1 mice group exhibited higher signal intensity on the fluorescence images, indicating an increase in tumor growth activity compared to the oe-NC group (Fig. 8D). The immunohistochemical staining results of the mice tumor tissue further validated observed expression alterations of METTL1, which were consistent with the findings from the in vitro cell experiments (Fig. 8E).

Following immunotherapy with CTLA4 or PD1 inhibitors, the analysis of survival rates in mice demonstrates a prolonged survival period in the sh-METTL1 + anti-IgG group compared to the sh-NC + anti-IgG group. Additionally, both the sh-METTL1 + anti-CTLA4 group and the oe-METTL1 + anti-PD1 group further prolong the survival period, suggesting that mice with silenced METTL1 exhibit an enhanced response to CTLA4 or PD1 inhibitor immunotherapy leading to increased survival. In contrast, compared to the oe-NC + anti-IgG group, mice in the oe-METTL1 + anti-IgG group exhibited a shorter survival period. However, there was no change in the survival period of mice in the oe-METTL1 + anti-CTLA4 or the oe-METTL1 + anti-PD1 group compared to the oe-METTL1 + anti-IgG group. These results indicate that mice overexpressing METTL1 had a poor response to CTLA4 or PD1 inhibitors and did not show improved survival rates (Fig. 8F).

In conclusion, studies on mouse models have shown that silencing METTL1 can inhibit tumor occurrence and development, increase mouse survival, and enhance the efficacy of immunotherapy targeting CTLA4 and PD1.

## Discussion

Interestingly, scRNA-seq technology has revolutionized our comprehension of tumor cellular heterogeneity, offering unprecedented insights into the complex biological landscape of cancers, including gastric cancer [20, 44, 45]. In this study, we have identified distinct subgroups within gastric cancer cells, with particular attention to groups 10 and 11, which manifest significantly elevated expression levels of genes closely associated with tumorigenesis [46, 47]. This stratification of tumor cells not only deepens our understanding of the intrinsic biological diversity within gastric cancer but also paves the way for the development of targeted therapeutic interventions.

In addition, our investigation shed light on the critical role of tRNA-related signaling pathways in the context of gastric cancer. The upregulation of the METTL1 gene within tumor specimens correlates with improved patient survival outcomes, suggesting that METTL1 may influence both the initiation and progression of gastric cancer [48]. The implications of our findings are profound, indicating that targeting METTL1 and its downstream signaling pathways could represent a viable strategy for the treatment of gastric cancer [49].

Further exploration into the regulatory dynamics of METTL1 has underscored its pivotal importance in gastric cancer pathophysiology. Experimental manipulation revealed that downregulation of METTL1 expression curtails the proliferation of gastric cancer cells while concurrently elevating apoptotic rates. Conversely, the forced overexpression of METTL1 augments cell proliferation, underscoring its central role in the modulation of gastric cancer cell behavior. Multiple studies have demonstrated that METTL1 promotes tumorigenesis through tRNA-derived fragment biogenesis in various cancer types [50–52]

We have developed a risk scoring framework based on METTL1-associated genes to enhance prognostic predictions for gastric cancer patients. This innovative system enables the stratification of patients into distinct risk categories, thereby facilitating the delivery of more tailored therapeutic recommendations by healthcare professionals. Notably, our risk assessment model underscores the critical influence of the tumor immune microenvironment on patient outcomes. Enhanced infiltration of immune cells within the tumors of patients categorized as high risk illuminates the intricate interplay between immune responses and the pathogenesis of gastric cancer [53].

The random forest model offers a novel approach to predicting the prognosis of patients with gastric cancer [21, 22], exhibiting superior predictive performance relative to conventional clinical metrics. The validation of our risk score as an autonomous prognostic indicator further emphasizes its utility in clinical settings [43]. This study contributes a novel, highly accurate tool for prognostic assessment, offering the potential to significantly improve patient management in gastric cancer, highlighting the transformative impact of integrating molecular and computational approaches in the fight against this formidable cancer [54].

## Conclusion

In conclusion, the present study, based on scRNA and a random forest model, explored the cellular heterogeneity inherent in gastric cancer, revealing distinct tumor cell subgroups with varying expression of tumor-related genes. The identification of the METTL1 gene as a key player in tRNA-related signaling pathways and its association with improved patient survival underscores the potential of METTL1 as a therapeutic target (Figure S4). The development of a risk scoring system based on METTL1-related genes for prognostic prediction represents a significant advancement in personalized medicine for gastric cancer patients. Despite these advancements, our study is not without limitations. The classification of tumor cells into subgroups and the identification of METTL1 as a therapeutic target are based on observational data, which may not fully capture the complex interactions within the tumor microenvironment. Additionally, the predictive accuracy of the risk scoring system, while promising, requires validation in larger, independent cohorts to confirm its clinical utility. Additionally, experimental studies aimed at elucidating the mechanistic role of METTL1 in gastric cancer progression and response to therapy are warranted.

### Supplementary Information

Below is the link to the electronic supplementary material.

Fig. S1. RT-qPCR detection of METTL1 expression and silencing efficiency in different MFC cell lines. *P < 0.05 compared with the sh-NC group; # *P* < 0.05 compared with the oe-NC group; Cell experiments repeated 3 times. 1 (JPG 127 KB)

Fig. S2. Correlation between risk scoring and immune cell infiltration in gastric cancer patients. (A) Heatmap showing differences in immune function between high-risk and low-risk groups. (B) Evaluation of scores in different groups. (C) Scores in different groups. (JPG 1103 KB)

Fig. S3. Association of risk scoring with immunotherapy response and expression of immune checkpoint genes in gastric cancer patients. (A) TIDE values in different groups. (B) Sensitivity to immunotherapy in different groups. (C) Relationship between risk scoring and different immune checkpoint genes. (JPG 809 KB)

Fig. S4. Molecular mechanism illustration of the impact of METTL1 on the responses to immunotherapy in gastric cancer. (JPG 1124 KB)

Supplementary file5 (DOCX 14 KB)

## Data Availability

The datasets generated during and/or analyzed during the current study are available from the corresponding author upon reasonable request.

## References

[1] Kratzer TB, Jemal A, Miller KD (2023). Cancer statistics for American Indian and Alaska Native individuals, 2022: including increasing disparities in early onset colorectal cancer. CA Cancer J Clin.

[2] Arnold M, Abnet CC, Neale RE (2020). Global burden of 5 major types of gastrointestinal cancer. Gastroenterology.

[3] Allemani C, Matsuda T, Di Carlo V (2018). Global surveillance of trends in cancer survival 2000–14 (CONCORD-3): analysis of individual records for 37 513 025 patients diagnosed with one of 18 cancers from 322 population-based registries in 71 countries. Lancet.

[4] Giaquinto AN, Miller KD, Tossas KY, Winn RA, Jemal A, Siegel RL (2022). Cancer statistics for African American/Black People 2022. CA Cancer J Clin.

[5] Jiang H, Yu X, Li N (2022). Efficacy and safety of neoadjuvant sintilimab, oxaliplatin and capecitabine in patients with locally advanced, resectable gastric or gastroesophageal junction adenocarcinoma: early results of a phase 2 study. J Immunother Cancer.

[6] Roy S, Kanda M, Nomura S (2022). Diagnostic efficacy of circular RNAs as noninvasive, liquid biopsy biomarkers for early detection of gastric cancer. Mol Cancer.

[7] Zhang Z, Wu H, Chong W, Shang L, Jing C, Li L (2022). Liquid biopsy in gastric cancer: predictive and prognostic biomarkers. Cell Death Dis.

[8] Smyth EC, Nilsson M, Grabsch HI, van Grieken NC, Lordick F (2020). Gastric cancer. Lancet.

[9] Riley RS, June CH, Langer R, Mitchell MJ (2019). Delivery technologies for cancer immunotherapy. Nat Rev Drug Discov.

[10] Zhang Y, Zhang Z (2020). The history and advances in cancer immunotherapy: understanding the characteristics of tumor-infiltrating immune cells and their therapeutic implications. Cell Mol Immunol.

[11] Abbott M, Ustoyev Y (2019). Cancer and the immune system: the history and background of immunotherapy. Semin Oncol Nurs.

[12] Kennedy LB, Salama AKS (2020). A review of cancer immunotherapy toxicity. CA Cancer J Clin.

[13] van den Bulk J, Verdegaal EM, de Miranda NF (2018). Cancer immunotherapy: broadening the scope of targetable tumours. Open Biol.

[14] Walcher L, Kistenmacher AK, Suo H (2020). Cancer stem cells-origins and biomarkers: perspectives for targeted personalized therapies. Front Immunol.

[15] Gavrielatou N, Doumas S, Economopoulou P, Foukas PG, Psyrri A (2020). Biomarkers for immunotherapy response in head and neck cancer. Cancer Treat Rev.

[16] Hanjani NA, Esmaelizad N, Zanganeh S (2022). Emerging role of exosomes as biomarkers in cancer treatment and diagnosis. Crit Rev Oncol Hematol.

[17] Wen R, Zhou L, Peng Z (2023). Single-cell sequencing technology in colorectal cancer: a new technology to disclose the tumor heterogeneity and target precise treatment. Front Immunol.

[18] Zhang J, Song C, Tian Y, Yang X (2022). Single-cell RNA sequencing in lung cancer: revealing phenotype shaping of stromal cells in the microenvironment. Front Immunol.

[19] López-Bueno R, Andersen LL, Koyanagi A (2022). Thresholds of handgrip strength for all-cause, cancer, and cardiovascular mortality: a systematic review with dose-response meta-analysis. Ageing Res Rev.

[20] Jovic D, Liang X, Zeng H, Lin L, Xu F, Luo Y (2022). Single-cell RNA sequencing technologies and applications: a brief overview. Clin Transl Med.

[21] Blanchet L, Vitale R, van Vorstenbosch R (2020). Constructing bi-plots for random forest: tutorial. Anal Chim Acta.

[22] Hu J, Szymczak S (2023). A review on longitudinal data analysis with random forest. Brief Bioinform.

[23] Zhao H, Jiang R, Zhang C, Feng Z, Wang X (2023). The regulatory role of cancer stem cell marker gene CXCR4 in the growth and metastasis of gastric cancer. NPJ Precis Oncol.

[24] Cobos FA, Panah MJN, Epps J (2023). Effective methods for bulk RNA-seq deconvolution using scnRNA-seq transcriptomes. Genome Biol.

[25] Wongvibulsin S, Wu KC, Zeger SL (2019). Clinical risk prediction with random forests for survival, longitudinal, and multivariate (RF-SLAM) data analysis. BMC Med Res Methodol.

[26] Kang SY, Heo YJ, Kwon GY, Kim KM (2022). Expression of CD274 mRNA measured by qRT-PCR correlates with PD-L1 immunohistochemistry in gastric and urothelial carcinoma. Front Oncol.

[27] Roozbehani M, Abdolmohammadi MH, Hamzeloo-Moghadam M, Irani S, Fallahian F (2021). Gaillardin, a potent sesquiterpene lactone induces apoptosis via down-regulation of NF-κβ in gastric cancer cells, AGS and MKN45. J Ethnopharmacol.

[28] Park JH, Seo JH, Jeon HY (2020). Lentivirus-mediated VEGF knockdown suppresses gastric cancer cell proliferation and tumor growth in vitro and in vivo. Onco Targets Ther.

[29] Guo H, Ha C, Dong H, Yang Z, Ma Y, Ding Y (2019). Cancer-associated fibroblast-derived exosomal microRNA-98–5p promotes cisplatin resistance in ovarian cancer by targeting CDKN1A. Cancer Cell Int.

[30] Wang Z, Liang X, Xiong A (2021). Helichrysetin and TNF-α synergistically promote apoptosis by inhibiting overactivation of the NF-κB and EGFR signaling pathways in HeLa and T98G cells. Int J Mol Med.

[31] Ma D, Wu Z, Zhao X (2023). Immunomodulatory effects of umbilical mesenchymal stem cell-derived exosomes on CD4^+^ T cells in patients with primary Sjögren's syndrome. Inflammopharmacology.

[32] Alizadeh-Fanalou S, Alian F, Mohammadhosayni M, Rahban D, Abbasi Ghasem Kheyli P, Ahmadi M (2020). Dysregulation of microRNAs regulating survivin in CD4+ T cells in multiple sclerosis. Mult Scler Relat Disord..

[33] Chen X, Gao A, Zhang F (2021). ILT4 inhibition prevents TAM- and dysfunctional T cell-mediated immunosuppression and enhances the efficacy of anti-PD-L1 therapy in NSCLC with EGFR activation. Theranostics.

[34] Fang W, Zhou T, Shi H (2021). Progranulin induces immune escape in breast cancer via up-regulating PD-L1 expression on tumor-associated macrophages (TAMs) and promoting CD8^+^ T cell exclusion [published correction appears in J Exp Clin Cancer Res. 2022 Mar 12;41(1):93]. J Exp Clin Cancer Res.

[35] Zhao S, Li P, Wang P (2019). Nurr1 promotes lung cancer apoptosis via enhancing mitochondrial stress and p53-Drp1 pathway. Open Life Sci..

[36] Liu L, Xie D, Xie H (2019). ARHGAP10 inhibits the proliferation and metastasis of CRC cells via blocking the activity of RhoA/AKT signaling pathway. Onco Targets Ther.

[37] Zhu G, Ye J, Huang Y (2016). Receptor-interacting protein-1 promotes the growth and invasion in gastric cancer. Int J Oncol.

[38] Ahern E, Harjunpää H, O'Donnell JS (2018). RANKL blockade improves efficacy of PD1-PD-L1 blockade or dual PD1-PD-L1 and CTLA4 blockade in mouse models of cancer. Oncoimmunology.

[39] Chan SM, Lin BF, Wong CS, Chuang WT, Chou YT, Wu ZF (2017). Levobuipivacaine-induced dissemination of A549 lung cancer cells. Sci Rep.

[40] Taromi S, Kayser G, von Elverfeldt D (2016). An orthotopic mouse model of small cell lung cancer reflects the clinical course in patients. Clin Exp Metastasis.

[41] Zong S, Dai W, Guo X, Wang K (2021). LncRNA-SNHG1 promotes macrophage M2-like polarization and contributes to breast cancer growth and metastasis. Aging (Albany NY).

[42] Zhang X, Wang Y, Gari A, Qu C, Chen J (2021). Pan-cancer analysis of PARP1 alterations as biomarkers in the prediction of immunotherapeutic effects and the association of its expression levels and immunotherapy signatures. Front Immunol.

[43] Li Y, Hu X, Lin R (2022). Single-cell landscape reveals active cell subtypes and their interaction in the tumor microenvironment of gastric cancer. Theranostics..

[44] Ziegenhain C, Vieth B, Parekh S (2017). Comparative analysis of single-cell RNA sequencing methods. Mol Cell.

[45] Slovin S, Carissimo A, Panariello F (2021). Single-cell RNA sequencing analysis: a step-by-step overview. Methods Mol Biol.

[46] Li R, Wu X, Wei H, Tian S (2013). Characterization of side population cells isolated from the gastric cancer cell line SGC-7901. Oncol Lett.

[47] Gao G, Sun Z, Wenyong L, Dongxia Y, Zhao R, Zhang X (2015). A preliminary study of side population cells in human gastric cancer cell line HGC-27. Ann Transplant.

[48] Ma X, Qiu S, Tang X (2022). TSPAN31 regulates the proliferation, migration, and apoptosis of gastric cancer cells through the METTL1/CCT2 pathway. Transl Oncol.

[49] Zeng Z, Zhang X, Jiang CQ (2022). Identifying novel therapeutic targets in gastric cancer using genome-wide CRISPR-Cas9 screening. Oncogene.

[50] García-Vílchez R, Añazco-Guenkova AM, Dietmann S (2023). METTL1 promotes tumorigenesis through tRNA-derived fragment biogenesis in prostate cancer. Mol Cancer.

[51] Gao Z, Xu J, Zhang Z (2022). A comprehensive analysis of METTL1 to immunity and stemness in pan-cancer. Front Immunol.

[52] Zeng X, Liao G, Li S (2023). Eliminating METTL1-mediated accumulation of PMN-MDSCs prevents hepatocellular carcinoma recurrence after radiofrequency ablation. Hepatology.

[53] Zeng D, Li M, Zhou R (2019). Tumor microenvironment characterization in gastric cancer identifies prognostic and immunotherapeutically relevant gene signatures. Cancer Immunol Res.

[54] Chang J, Wu H, Wu J (2023). Constructing a novel mitochondrial-related gene signature for evaluating the tumor immune microenvironment and predicting survival in stomach adenocarcinoma. J Transl Med.

