# Supplementary figures and images for: New genetic insights into immunotherapy outcomes in gastric cancer via single-cell RNA sequencing and random forest model
Source: Cancer Immunol Immunother. 2024 May 2;73(6):112. doi: 10.1007/s00262-024-03684-8 (PMC11063021; doi:10.1007/s00262-024-03684-8)

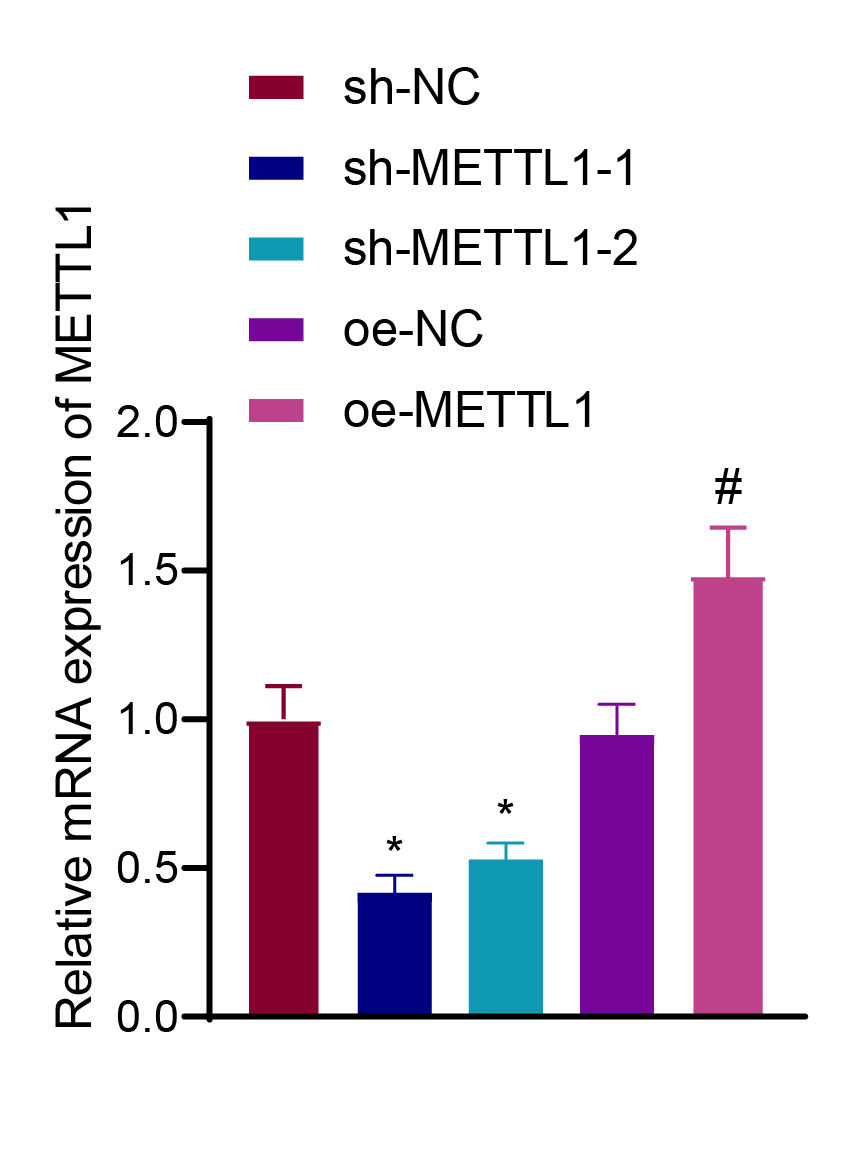

Supplement: Supplementary file 1 — Fig. S1. RT-qPCR detection of METTL1 expression and silencing efficiency in different MFC cell lines. *P < 0.05 compared with the sh-NC group; # P < 0.05 compared with the oe-NC group; Cell experiments repeated 3 times. 1 (JPG 127 KB) [file 262_2024_3684_MOESM1_ESM.jpg]

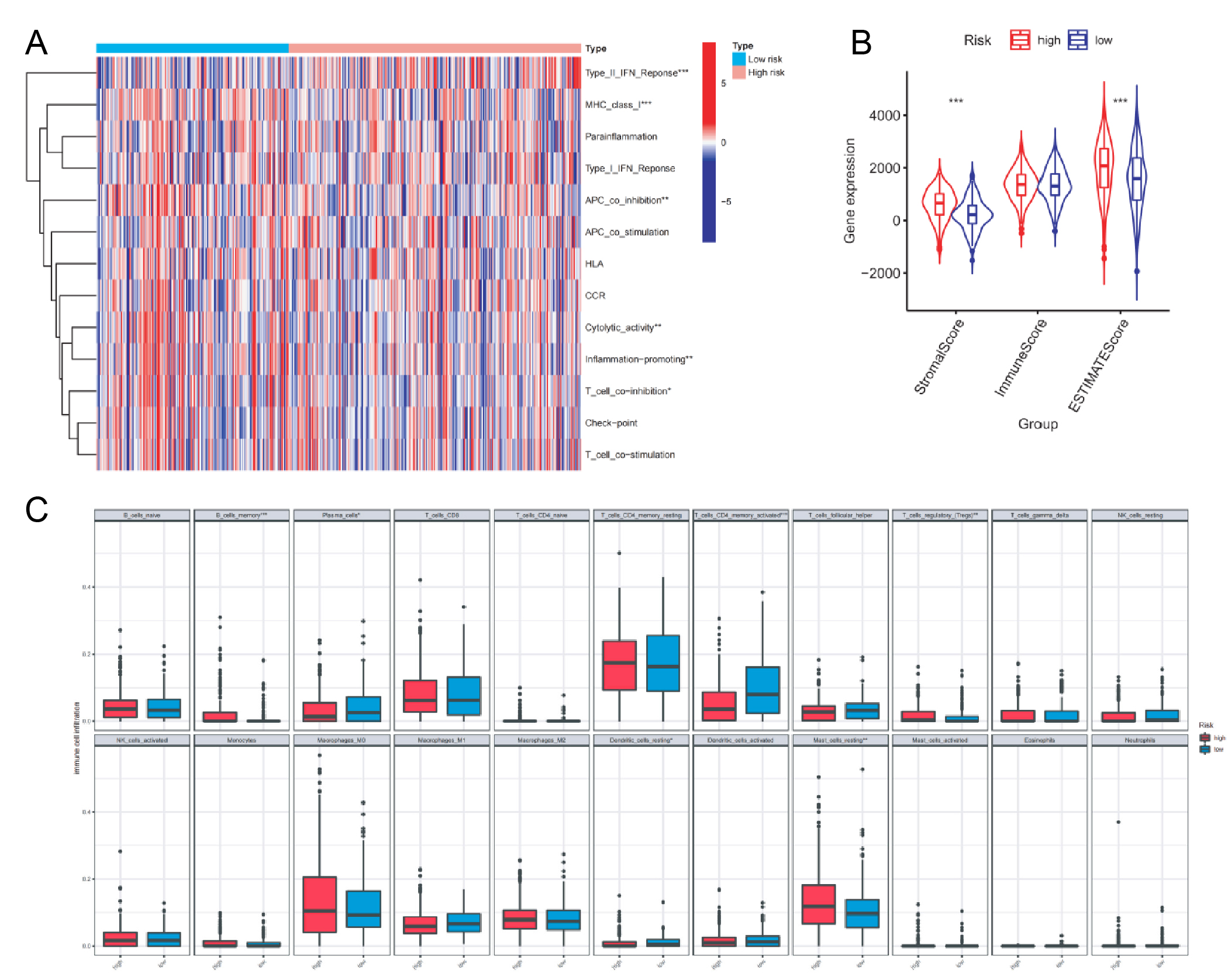

Supplement: Supplementary file 2 — Fig. S2. Correlation between risk scoring and immune cell infiltration in gastric cancer patients. (A) Heatmap showing differences in immune function between high-risk and low-risk groups. (B) Evaluation of scores in different groups. (C) Scores in different groups. (JPG 1103 KB) [file 262_2024_3684_MOESM2_ESM.jpg]

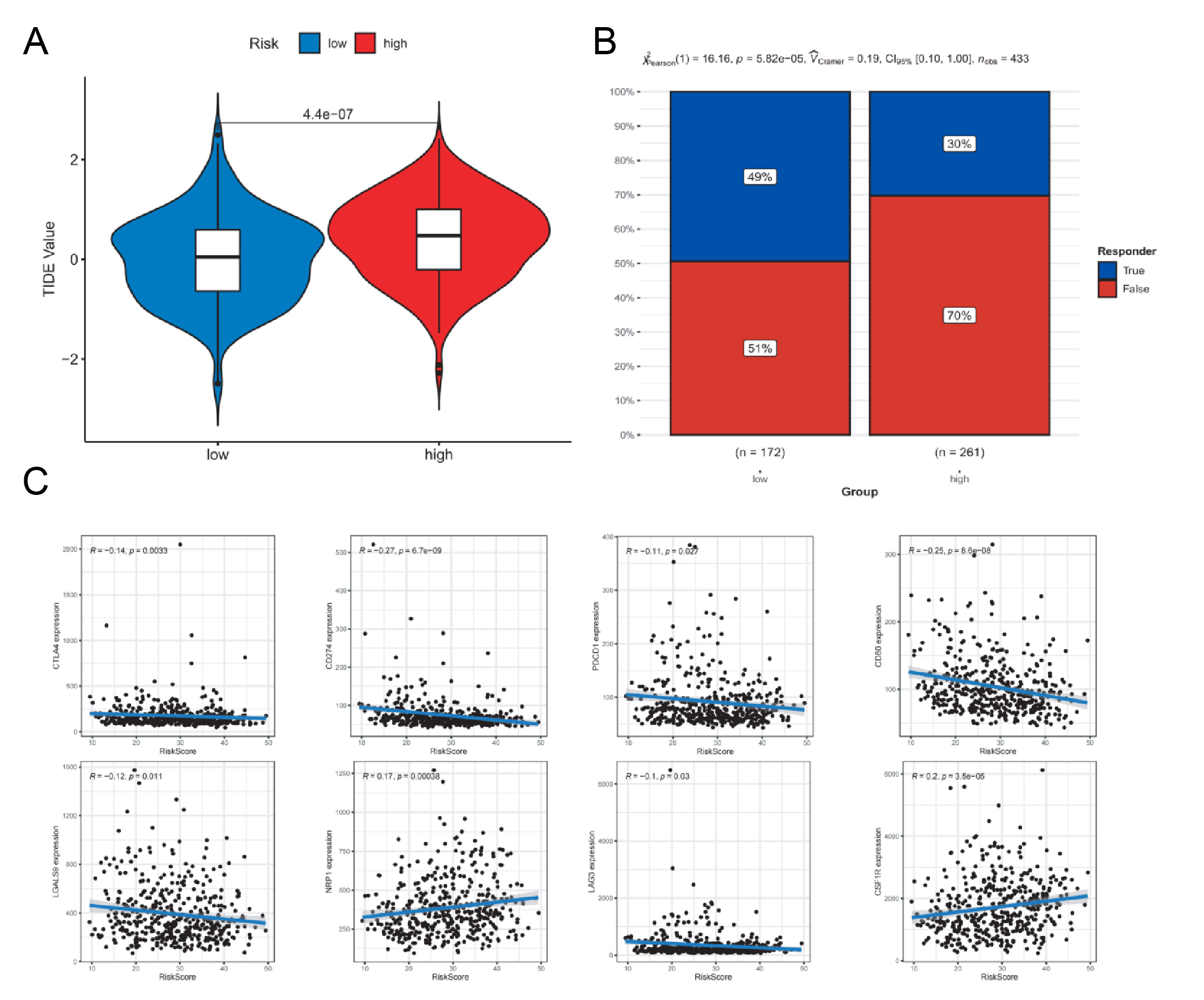

Supplement: Supplementary file 3 — Fig. S3. Association of risk scoring with immunotherapy response and expression of immune checkpoint genes in gastric cancer patients. (A) TIDE values in different groups. (B) Sensitivity to immunotherapy in different groups. (C) Relationship between risk scoring and different immune checkpoint genes. (JPG 809 KB) [file 262_2024_3684_MOESM3_ESM.jpg]

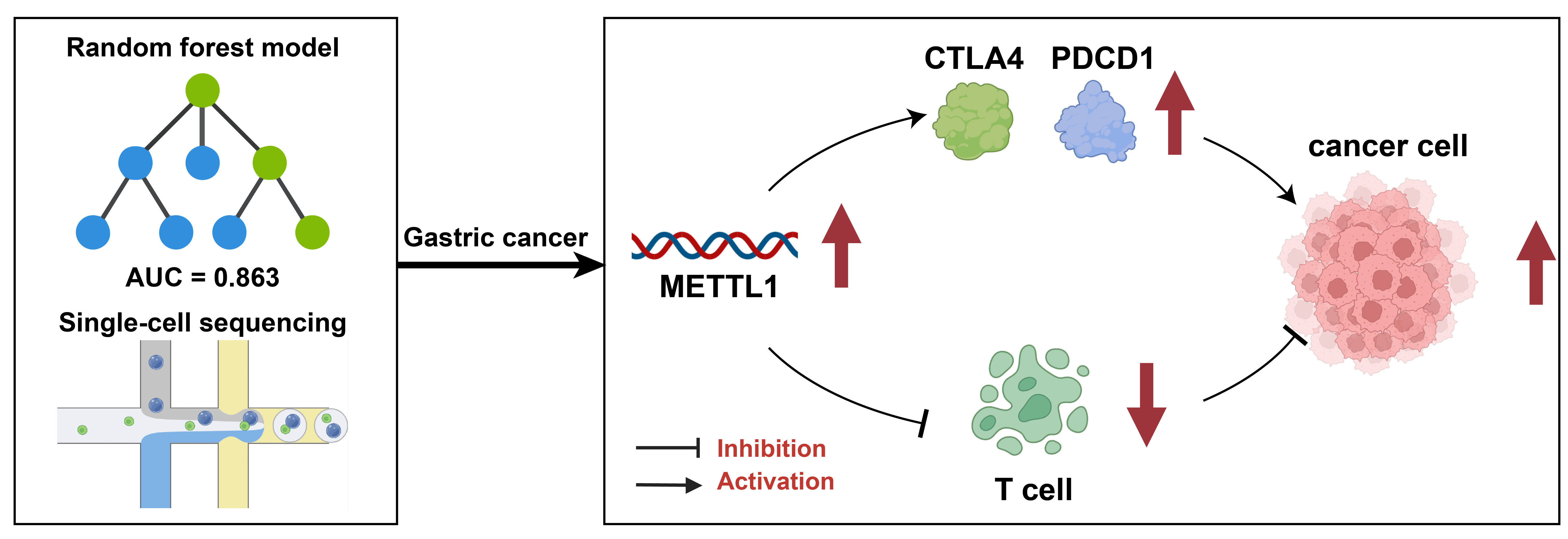

Supplement: Supplementary file 4 — Fig. S4. Molecular mechanism illustration of the impact of METTL1 on the responses to immunotherapy in gastric cancer. (JPG 1124 KB) [file 262_2024_3684_MOESM4_ESM.jpg]
