# Supplementary material for: New genetic insights into immunotherapy outcomes in gastric cancer via single-cell RNA sequencing and random forest model
Source: Cancer Immunol Immunother. 2024 May 2;73(6):112. doi: 10.1007/s00262-024-03684-8 (PMC11063021; doi:10.1007/s00262-024-03684-8)
Supplement: Supplementary file 5 — Supplementary file5 (DOCX 14 KB) [file 262_2024_3684_MOESM5_ESM.docx]

**Table S1. RT-qPCR primer sequences**

| Gene | Forward (5’-3’) | Reverse (5’-3’) |
| --- | --- | --- |
| METTL1 (mouse) | ACCCAGAGTTCTTTGCTCCG | TACACCAGGCCCCCGACT |
| METTL1 (human) | CGCTACCCTGTGAAGCCAG | GCTTCATGGCATTGCTACGG |
| GAPDH (mouse) | GAAGGTCGGTGTGAACGGAT | ACTGTGCCGTTGAATTTGCC |
| GAPDH (human) | AATGGGCAGCCGTTAGGAAA | TTCCCGTTCTCAGCCTTGAC |

**Table S2. shRNA sequences**

| shRNA | Sequence |
| --- | --- |
| sh-NC | 5’-TTCTCCGAACGTGTCACGT-3’ |
| sh-METTL1-1 (human) | 5’-CCATGTACTTACAAGCCGATA-3’ |
| sh-METTL1-2 (human) | 5’-GCCGTGGACGAGAAAGAAATA-3’ |
| sh-METTL1-1 (mouse) | 5’-CGCCATGAAACACCTTCCTAA-3’ |
| sh-METTL1-2 (mouse) | 5’-CCCACACTTTAAGCGAACGAA-3’ |
